# Bioengineering of Periodontal Tissues: Cell Therapy and Biomaterials Application

**DOI:** 10.3390/bioengineering12111213

**Published:** 2025-11-06

**Authors:** Mohammad Hadi Norahan, Sudesh Sivarasu, Alexey Fayzullin, Chibuike Mbanefo, Polina Bikmulina, Igor Ashurko, Iana Khristidis, Peter Timashev

**Affiliations:** 1 Institute for Regenerative Medicine, I.M. Sechenov First Moscow State Medical University (Sechenov University), 8/2 Trubetskaya St., Moscow 119991, Russia; norakhan_m_kh@staff.sechenov.ru (M.H.N.); timashev_p_s@staff.sechenov.ru (P.T.); 2 Department of Human Biology, University of Cape Town, Cape Town 7701, Western Cape, South Africa; sudesh.sivarasu@uct.ac.za (S.S.); chibuike.mbanefo@uct.ac.za (C.M.)

**Keywords:** periodontal regeneration, stem cell therapy, tissue engineering, biomaterials, 3D bioprinting, biofabrication

## Abstract

Periodontal regeneration remains one of the most demanding challenges in oral bioengineering due to the structural complexity of the periodontium and the inflammatory microenvironment accompanying disease. Conventional surgical and pharmacological therapies often fail to achieve full restoration of bone, ligament and cementum, prompting the development of cell-based and biomaterial-assisted approaches. This review summarizes current advances in cellular technologies for periodontal regeneration, emphasizing the biological rationale, material design and delivery methods shaping next-generation treatments. We discuss stem-cell-based strategies employing periodontal ligament, dental pulp and mesenchymal stem cells, their paracrine and immunomodulatory roles, and how their therapeutic potential is enhanced through integration into engineered scaffolds. Recent progress in hydrogel systems, microspheres, decellularized matrices and 3D bioprinting is analyzed, highlighting how structural cues, bioactive nanoparticles and gene-modified cells enable multi-tissue regeneration. Emerging delivery and biofabrication techniques, from manual seeding to automated and in situ printing, are reviewed as key determinants of clinical translation. The convergence of bioprinting precision, immune-responsive biomaterials and personalized cellular constructs positions periodontal bioengineering as a rapidly maturing field with strong prospects for functional restoration of diseased oral tissues.

## 1. Introduction

Periodontological diseases represent a significant public health challenge, primarily due to their role as a leading cause of tooth loss among adults globally. Periodontitis, a severe form of periodontal disease characterized by chronic bacterial infections and inflammatory destruction of periodontal tissues, substantially impacts oral health and quality of life. Retrospective studies have highlighted that periodontal diseases account for approximately 61.8% of tooth loss cases [1]. Longitudinal data further reinforce this concern, indicating that nearly 10% of teeth in adults are lost directly due to periodontitis [2]. Such substantial tooth loss contributes to various difficulties, including compromised nutrition, reduced oral function and social isolation, highlighting the critical importance of preventive and therapeutic interventions [3].

Globally, periodontal diseases affect up to 50% of adults, with severe forms of periodontitis observed in approximately 5–10% of the adult population [4]. Gingivitis, a milder reversible form characterized by gum inflammation without deeper tissue destruction, is nearly ubiquitous, occurring in almost every individual at some point in their lives [5]. The prevalence of periodontal diseases varies notably by region, age and socioeconomic status. Higher-income countries generally exhibit lower prevalence rates due to better access to preventive and therapeutic dental care. Conversely, lower socioeconomic groups and populations in low- and middle-income countries suffer disproportionately from severe periodontal disease due to limited healthcare access, inadequate oral hygiene education and lifestyle factors such as poor nutrition and increased tobacco and alcohol consumption [6,7,8,9].

The economic burden of periodontitis is substantial, exerting significant financial pressure on healthcare systems worldwide. Treatment costs and indirect economic impacts, such as lost productivity due to oral health problems, reach staggering levels. For instance, the economic costs associated with managing periodontal diseases were estimated at approximately €149.52 billion in Europe and €122.65 billion in the United States in 2018 [10]. Additionally, in Malaysia, periodontitis-related expenses accounted for around 3.83% of the nation’s GDP [11]. Given these figures, addressing periodontal diseases through improved screening, preventive measures and effective early intervention strategies is vital not only to reduce healthcare expenditures but also to enhance the overall health and productivity of populations worldwide.

Contemporary therapeutic approaches for periodontal diseases primarily focus on mechanical debridement methods, including scaling and root planing, which aim to remove bacterial biofilms and calculus from tooth surfaces and periodontal pockets [12]. Adjunctive therapies such as systemic or local antibiotic applications and antiseptic mouth rinses are frequently employed to enhance treatment outcomes and manage persistent infections [13]. However, these approaches face several limitations. Mechanical therapy often fails to fully eradicate pathogens embedded deeply within periodontal tissues, leading to frequent disease recurrence [14]. Antibiotic therapies pose risks of developing resistant bacterial strains and may cause systemic side effects [15]. Moreover, both mechanical and antibacterial therapies do not guide structural restoration of the periodontal tissues, causing destruction of this niche responsible for tooth metabolism and regeneration.

Due to limitations of conventional therapies to provide a complete regeneration for periodontal defects, cell therapy and tissue engineering have been extensively studied as an alternative therapy [16]. Cell therapy recruits different cell types (e.g., periodontal ligament stem cells, mesenchymal stem cells) to restore damaged tissue through different mechanisms [17]. Tissue engineering is a modern multidisciplinary field that integrates different specialties to regenerate functional tissues and organs affected by damage or disease [18]. Tissue engineering involves using biomaterial-based structures containing cells and bioactive agents to provide a biomimetic microenvironment for tissue regeneration [19]. In this review, we explore how cell therapy and cell-containing structures are improving periodontal tissue regeneration. First, we discuss cell therapy, where the cells are directly delivered to the periodontal defect site. Then, we focus deeply on biomaterial-assisted cell transplantation, examining cell-containing scaffolds: three-dimensional structures, typically composed of biopolymers, that serve as “homes” for cells, promoting their proliferation and aiding in the regeneration of periodontal defects, along with other techniques of biomaterial-mediated cell transplantation for periodontal regeneration. Finally, we delve into the problem of delivery devices required for the wide implementation of bioengineering in dentistry.

## 2. Cell Therapy for Periodontal Regeneration

Conventional therapies for craniofacial and periodontal defects, including bone grafts and guided tissue regeneration with barrier membranes, are limited by their inability to fully restore the native physiological and anatomical structures. While these approaches can promote some bone fill and soft tissue attachment, they often fail to recapitulate the complex, highly organized architecture of the periodontium, particularly the perpendicularly oriented collagen fibers of the periodontal ligament (PDL) that provide robust biomechanical function. Furthermore, commonly used materials, such as non-resorbable membranes, are often biologically inert, acting merely as passive barriers rather than actively promoting integrated regeneration. Consequently, the outcome is often a repair process characterized by an epithelium and a functionally inferior cementum with poor tissue connectivity, rather than a true regeneration of the lost tissues. Therefore, there is a constant need in for more advanced solutions, such as cell-based therapy.

**Figure 1:**
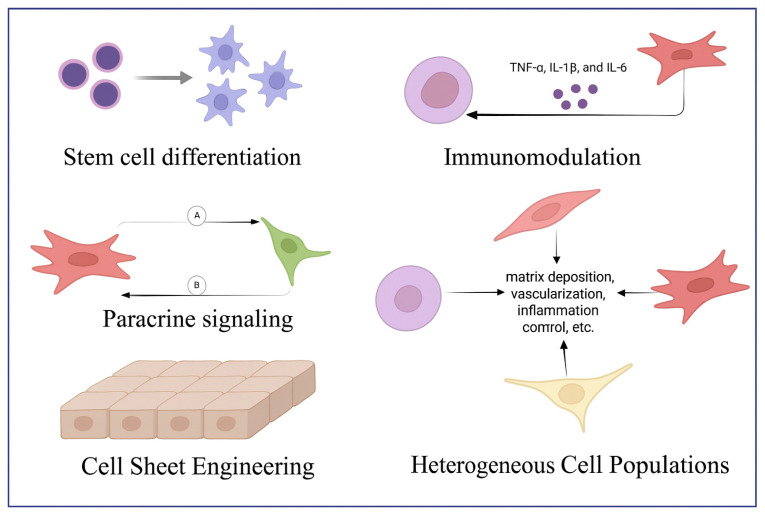
Mechanisms of cell therapy pro-regenerative effects on periodontal tissues. A and B represent signaling molecules acting in neighboring cells.

Cell therapy is a therapeutic strategy that involves the administration of autologous (sourced from the patient), allogeneic (sourced from a donor) or xenogenic (sourced from another species) cells to treat or repair damaged tissues in patients. Cell therapy utilizes various cell types, including stem cells and non-stem cells, for tissue regeneration or other therapeutic purposes, employing either homogeneous or heterogeneous cell populations [20]. It recruits cells, with or without genetic modifications or manipulations in formulations, which could be delivered to the target site through different methods such as injection or infusions [20,21]. Cell therapy can facilitate tissue regeneration through the differentiation potential and paracrine signaling mechanisms of stem cells [20,22]. Secretion of growth factors, cytokines and chemokines by mesenchymal stem cells (MSCs) and adult stem cells (ASCs) enhances angiogenesis, alleviates inflammation and activates endogenous progenitor cells to re-establish tissue homeostasis [22]. Therapies using heterogeneous cell populations such as bone marrow aspirate concentrate (BMAC) and stromal vascular fraction (SVF) utilize synergistic interactions in mixed cellular populations, incorporating hematopoietic stem cells, endothelial progenitors and immune cells to replicate natural tissue repair mechanisms [20]. These therapeutic approaches promote tissue regeneration via cell–cell interaction and extracellular matrix remodeling, vital for the regenerative environment of damaged tissue [20]. Because of their exceptional capacity for self-renewal, stem cells, as the foundational cells and the building blocks of all organs and tissues, can divide to generate new cells of any kind, thereby contributing to the healing of damaged tissue [23]. The healthy periodontal ligament (PDL) has a limited capacity for regeneration and retains stem-cell niches throughout adulthood. But in a diseased periodontal environment, the lack of healthy stem cells makes the use of ex vivo expanded/manipulated stem cells necessary [23]. In this case, periodontal cell therapy aims to improve the regeneration process and restore the periodontium’s structural and functional integrity by introducing new cells, such as dental pulp stem cells (DPSCs), periodontal ligament stem cells (PDLSCs) and MSCs, into a defect site [24]. Instead of directly contributing to tissue regeneration, the transplanted cells may either regulate repair through the secretion of growth or cellular signals, or they may function as building blocks by differentiating into multiple cell types and aiding in the repair of damaged periodontium [23]. By directly interacting with cellular processes like differentiation, immunomodulation and paracrine signaling, cell therapy addresses the underlying cause of tissue loss, in contrast to traditional treatments that frequently fail to succeed in full regeneration (Figure 1) [25].

Stem cells can regenerate the various tissues of the periodontium because of their exceptional differentiation plasticity [25]. For example, PDLSCs exhibit a special ability to differentiate into periodontal ligament fibroblasts, osteoblasts and cementoblasts, all of which are essential for reconstructing the tooth-supporting structure [26,27]. Transplanted PDLSCs have been demonstrated in preclinical models to produce cementum-specific proteins, including cementum protein-1 (CEMP1) and cementum attachment protein (CAP), while also delivering mineralized matrix to restore alveolar bone defects [27]. Likewise, DPSCs aid in the regeneration of the dentin-pulp complex and can transform into functional cells resembling cementoblasts in response to particular microenvironmental cues, creating reparative cementum on exposed root surfaces [28,29]. MSCs derived from bone marrow (BMSCs) also promote osteogenesis by transforming into osteoblasts and releasing bone morphogenetic proteins (BMPs), which accelerates the mineralization of alveolar bone. Runx2 and Osterix are transcriptional factors that tightly regulate this multilineage potential and are upregulated during stem cell-mediated tissue regeneration [27].

In addition to directly differentiating, stem cells use paracrine signaling to coordinate regeneration by releasing bioactive compounds that regulate nearby cells and tissues [30]. Growth factors secreted by stem cells, such as platelet-derived growth factor (PDGF), fibroblast growth factor (FGF) and vascular endothelial growth factor (VEGF), promote angiogenesis, activate endogenous progenitor cells and improve the synthesis of extracellular matrix [31]. In regenerating periodontal tissues, for instance, VEGF secreted by DPSCs can increase microvascular density, providing a sufficient supply of nutrients for healing. DPSCs can directly contribute to angiogenesis and differentiate into cells that resemble endothelium. They can also function as pericytes, secreting growth factors like fibroblast growth factor 2 (FGF-2), PDGF and insulin-like growth factor 1 (IGF-1) to support blood vessel formation, stabilization and maturation, along with mediating the action of VEGF on endothelial cells (EC) to promote angiogenesis [32]. Bone marrow mesenchymal stem cell-derived small extracellular vesicles (BMSC-sEVs) have been found to increase the levels of tumor susceptibility gene 101 (TSG101) and the exosome protein CD63, which may help human periodontal ligament cells (hPDLC) migrate, grow and form bone. The BMSC-sEVs were effective in treating periodontitis through reduction in alveolar bone loss, inflammatory infiltration and collagen destruction in the rat periodontitis model [33]. The complex role that stem cells play in coordinating both structural repair and functional restoration is highlighted by these paracrine interactions.

Treatment approaches that make use of diverse cell populations, like SVF or bone marrow aspirate concentrate (BMAC), leverage the synergistic interactions among mesenchymal, endothelial and hematopoietic lineages [20]. With its hematopoietic stem cells (HSCs), endothelial progenitors and MSCs, BMAC mimics the cascade of natural repair by addressing matrix deposition, vascularization and inflammation all at once [20]. HSCs in the bone marrow stimulate bone remodeling/repair through dynamic interactions with stromal cells, including inflammatory cells, endothelial cells and niche cells such as MSCs and Schwann cells, mediated by reciprocal molecular crosstalk [34,35]. Through cell-contact-dependent mechanisms such as Wnt/Notch signaling, SCF (stem cell factor) secretion and the chemokine CXCL12, MSCs preserve HSC function [35,36]. Moreover, MSCs produce extracellular matrix (ECM) proteins such as fibronectin and collagen that regulate HSC homing, self-renewal and mobilization [37]. It has also been shown that the vascular endothelial growth factor released by transplanted HSCs reduces both bone formation and the growth of blood vessels when blocked with a soluble antagonist, showing the importance of the paracrine mechanism [37]. These results highlight the synergistic interdependence of diverse cell populations in tissue regeneration and emphasize the importance of cell-to-cell contact and paracrine signaling among adjacent cells for the regenerative potential of stem cells.

One of the main characteristics of periodontal disease is chronic inflammation, which causes tissue destruction by overproduction of pro-inflammatory cytokines like TNF-α, IL-1β and IL-6 [38]. Stem cells have strong immunomodulatory effects that regulate this adverse environment [39]. IL-10 and TGF-β are two examples of anti-inflammatory mediators secreted by MSCs that suppress hyperactive immune responses by preventing macrophage polarization toward pro-inflammatory M1 phenotypes [40]. Furthermore, they stimulate the expansion of regulatory T-cells (Tregs), which reduce inflammation triggered by Th17 and avoid damage to surrounding tissues [41]. Additionally, a study demonstrated that PDLSC transplantation decreased gingival TNF-α levels by 60% and IL-1β by 45% in a canine model of experimental periodontitis, alongside considerable reductions in clinical attachment loss and bone resorption [42]. It was also demonstrated that xenogenous DFSCs promoted periodontal regeneration by stimulating neutrophil polarization toward the N2 phenotype (characterized by elevated CXCR4/VEGF and reduced NOS2/TNF-α) during early repair, enhancing innate immune-mediated tissue clearance and matrix remodeling [43]. They subsequently induce M2 macrophage polarization (↑CD163/CD86 ratio) and suppress osteoclastogenesis. The dual-phase immunoregulation, mediated by paracrine cues and direct cell interactions, accelerates the healing of periodontal defects by controlling inflammation and establishing a pro-regenerative microenvironment [43].

The delivery of stem cells has been improved by recent developments in cell sheet engineering, which preserve the cells’ natural ECM and cell–cell connections, enabling scaffold-free transplantation [44]. PDLSC, BMSC or alveolar periosteal cell (APC) sheets can be harvested intact using temperature-responsive culture dishes, preserving biological integrity without enzymatic digestion and retaining the integrins, fibronectin and collagen type I necessary for host tissue integration [44,45]. Potential for periodontal-implant integration was highlighted by the formation of cementum and PDL-like tissues surrounding implants after hPDLC sheets were transplanted into mandibular defects [46]. In another study, genetically engineered cell sheets outperformed cell injection techniques in induced periodontitis lesions, exhibiting notable alveolar bone and PDL regeneration [47]. According to these findings, cell sheets improve survival rates and functional outcomes by preserving cell polarity and extracellular matrix architecture. This makes them a promising regenerative strategy that delivers functional cells with preserved microenvironmental cues [44].

Despite the significant potential of cell therapy for periodontal regeneration, several considerations remain. The optimal cellular sources for conducting preclinical and clinical studies remain a topic of debate [48]. Furthermore, the immune system’s response remains a challenge that may require intervention through genetically modified cells [47]. A significant limitation of utilizing cells independently is their lack of durability and consistency at the defect site. Cellular suspensions alone lack integration with the host tissue, which reduces the cell grafting rate to the host tissue, leading to poor vascularization and interconnection inside the defect sites [49]. This significant problem may be alleviated to some extent through the use of bioengineered constructs, in which cells are integrated into bioactive scaffolds to recreate a tissue-like structure.

## 3. Biomaterial-Assisted Cell Transplantation for Periodontal Regeneration

Tissue engineering consists of three main elements: cells, biocompatible scaffolds and bioactive agents/growth factors. These principles simply indicate that to use the regenerative potential of living cells, we need to provide them a favorable microenvironment to grow and regenerate target tissue. Cell therapy has limitations that could be mitigated by employing biomaterials as supportive and complementary components to enhance cell-mediated tissue regeneration. Cell retention and survival are essential for cells to function as regenerative elements. Injecting cells alone in the defect sites has a relatively low regenerative capacity due to dispersal or quick death caused by lack of anchorage, hostile host environments (e.g., inflammation, hypoxia) or mechanical stress. The tissue engineering solution is to provide a safe and competent microenvironment for the cells, serving as a 3D matrix that enhances cell retention and survival by mimicking the native ECM. In addition, a 3D biomaterial-based environment can offer a framework that cells can utilize as scaffolds to create the desired tissue’s building blocks, which pure cell therapy approaches do not provide this mechanical support either. Moreover, bioengineered 3D scaffolds enhance the integration of resident cells with host tissue by ensuring proper adhesion, providing porosity to promote host cell infiltration, inducing vascularization at the graft site and exposing bioactive components that improve tissue regeneration. Immunogenicity could also be another concern for cell therapy, where using allogeneic cells can cause the risk of immune rejection and the need for immunosuppressives. In tissue engineering strategy, however, 3D scaffolds can be either made of immune-shielding scaffold materials such as decellularized ECM or contain inflammatory/immunomodulatory agents to resolve harmful inflammation while preserving the immune system’s protective and regenerative functions.

A wide range of biomaterials with different structural and biological properties are known for periodontal regeneration, functioning either as the main polymer matrix or as functional additives [50]. The polymeric matrix, which helps with structural stability and engraftment and stops the gingival epithelium from growing down, is made up of biopolymers and synthetic polymers [51]. Collagen is a key biopolymer for replicating periodontium tissues helping biomineralization [50]. Gelatin offers similar cell-adhesive motifs (e.g., RGD sequences) and its more processable derivative, gelatin methacryloyl (GelMA), are used for PDL repair and alveolar bone formation [52,53,54]. Polysaccharides are also extensively utilized; chitosan is beneficial due to its antimicrobial and anti-inflammatory properties [55]. Hyaluronic acid (HA) is known for its hydrophilicity and viscoelasticity [56]. Alginate is cost-effective but often needs to be combined with other materials to improve bioactivity and its mechanical properties [57]. Synthetic polymers such as polycaprolactone (PCL), polylactic-polyglycolic acid (PLGA), and polyethylene glycol (PEG), while biocompatible and biodegradable, provide improved mechanical stability and processability; however, they do not possess natural cell-adhesiveness, necessitating modifications for optimal application [58]. Additives are added to the matrix to make improvements in terms of structure or biology. These include antibacterial/antioxidant agents (e.g., curcumin, quercetin, silver nanoparticles) to combat infection and oxidative stress [59,60]; growth factors (e.g., bFGF, PDGF, VEGF) to regulate cellular recruitment and angiogenesis [31,61,62]; nanoparticles such as metallic (e.g., AuNPs, nMgO) and carbon-based (e.g., graphene oxide, carbon nanotubes) types for antibacterial action, immunomodulation, and osteogenic induction [63,64,65,66]; and inorganic additives like hydroxyapatite (HAp), β-tricalcium phosphate (β-TCP), calcium sulfate (CS), and bioactive glass to improve mechanical strength, osteointegration, and AB regeneration [67,68].

**Figure 2:**
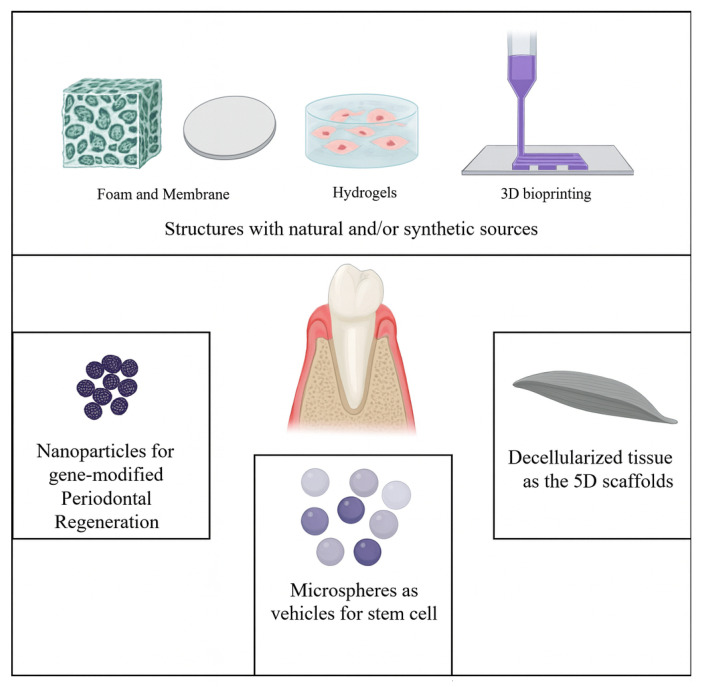
Diverse strategies employing biomaterials integrated with cells for periodontal regeneration.

In this section of this review paper, we highlight recent studies that focus on using cell-containing 3D structures for periodontal regeneration, specifically regarding their shape or fabrication methods in the past five years (Figure 2). We will discuss how the results of these recent studies show promise for achieving proper regeneration of periodontal defects. Moreover, we will discuss other types of biomaterial-assisted cell transplantation strategies for periodontal regeneration. Due to the large number of studies, we discuss and consider the ones that directly assess the regenerative capacity of the engineered structures in periodontal defect models (Table 1).

### 3.1. Freeze-Dried, Foam and Membrane Structures

One of the simpler types of scaffolds for tissue engineering applications is freeze-dried porous structures. This technique is based on casting the polymeric solution and using various potential mechanisms to induce polymerization, followed by a lyophilization procedure to achieve porous sponge-shaped scaffolds. During the past few decades, hundreds of studies have been conducted on the application of these sponge-shaped scaffolds to engineer structures for various tissue regeneration [92,93,94,95,96,97,98,99,100,101]. In addition to being simple and affordable to fabricate, these scaffolds could be engineered using a broad range of polymers and solutions and additives, minimizing the limitations of more sophisticated approaches like bioprinted or in situ forming hydrogels [93]. Freeze-dried scaffolds could usually possess structures with porosity in a range of 70–95% and pore size of 50–500 µm, which is favorable for infiltration and interconnection of mammalian cells’ network, nutrient transport and vascularization [92,102].

Calcium Phosphate (CaP) bioceramics are one of the most commonly used bio-materials in the field of osteogenic regeneration because they have a similar composition to bone mineral and are biocompatible, biodegradable, osteoinductive, and osteoconduc-tive [67]. Furthermore, CaP salts can create mineralized tissues, making them a suitable choice for endodontic therapy. Moreover, ceramics have become a very promising type of material for use as tissue adhesives in periodontal regeneration. Some ceramics, like bioactive glasses and calcium phosphate cement, can form a direct chemical bond with both hard and soft tissues. This makes a stable and integrated seal. This method is different from many adhesives that can be cytotoxic or just act as passive barriers [103].

**Figure 3:**
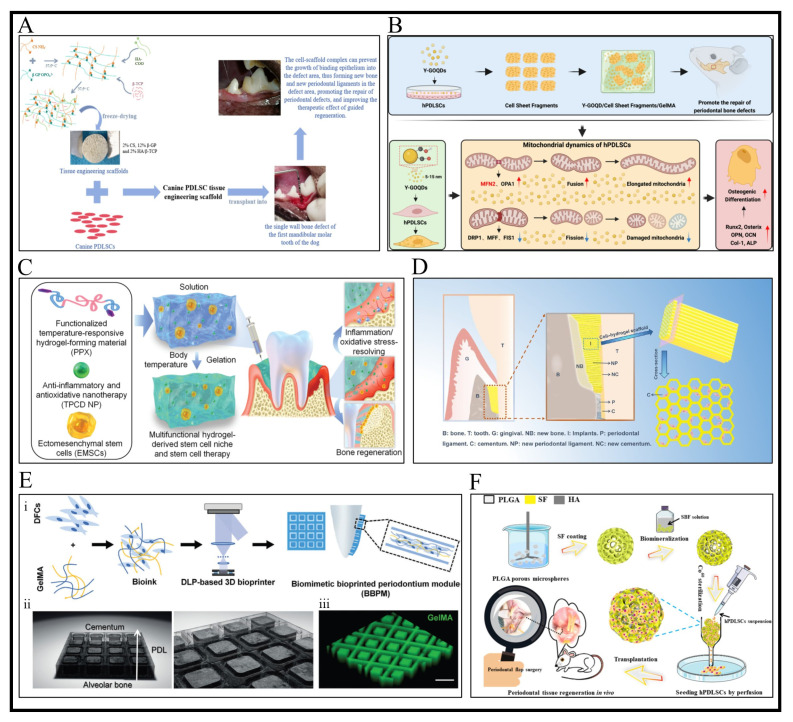
Different approaches for cell-containing biomaterial-assisted guided regeneration of periodontal tissue. (**A**) The design involves a freeze-dried chitosan structure combined with β-glycerol phosphate (β-GP) and biphasic calcium phosphate HA/β-TCP, which is seeded with periodontal ligament stem cells (PDLSCs) to promote periodontal tissue regeneration in canine mandibular bone defects. Reprinted and adapted with permission from Dai et al., 2024 [69]. (**B**) GelMA hydrogels incorporating hPDLSC sheets with two distinct varieties of graphene oxide quantum dots (GOQDs) were employed to address a periodontal defect model in rats. Red arrows indicate promoting fusion and blue arrows preventing fission in mitochondrial dynamics of hPDLSCs. Reprinted and adapted with permission from An et al., 2024 [75]. (**C**) Development of a multifunctional hydrogel with temperature-responsive in situ gelation properties as a niche for ectomesenchymal stem cells (EMSCs) in the treatment of periodontitis. Reprinted and adapted with permission from Li et al., 2023 [107]. (**D**) Development of an aligned porous cell containing a hydrogel scaffold based on chitosan (CS) and oxidized chondroitin sulfate (OCS) using the freeze-casting technique to induce the arrangement of periodontal tissue regeneration. Reprinted and adapted with permission from Wang et al., 2023 [79]. (**E**) Development of biomimetic periodontium patches (BPPs) containing dental follicle cells (DFCs) using microscale continuous digital light projection (mCDLP) bioprinting. (i) Schematic representation of BPP fabrication with a DLP-based 3D bioprinter. (ii) Schematic representation of the BPP. (iii) BPPs tagged with green fluorescence. Scale bar: 200 μm. Reprinted and adapted with permission from Ma et al., 2023 [82]. (**F**) Fabrication of PLGA porous microspheres (PMs) coated with silk fibroin (SF) and hydroxyapatite (HA) for the delivery of hPDLSCs utilizing a “cell perfusion” technique. Reprinted and adapted with permission from Liu et al., 2021 [90].

A chitosan (CS) freeze-dried structure incorporating β-glycerol phosphate (β-GP) and biphasic calcium phosphate (HA/β-TCP) was recently developed as a scaffold for periodontal ligament PDLSC aimed at regenerating periodontal tissue in canine mandibular bone defects (Figure 3A) [69]. CS is a biodegradable and biocompatible polymer derived from chitin, which, due to its strong adhesion and antibacterial qualities, is frequently used in wound healing research [104]. The optimal combination of the materials for the scaffolds was 2% CS, 12% β-GP and 2% HA/β-TCP with pore diameters between 300 and 600 μm. Additionally, the scaffold’s porosity balanced its compressive strength (0.44 ± 0.09 MPa), giving it a certain mechanical strength, preserving its structure and allowing it to withstand pressure from the surrounding tissues. While HA/β-TCP improved surface roughness and compressive strength, β-GP crosslinking with CS created a honeycomb pore structure that balanced mechanical stability and interconnected porosity, which led to these results [69]. Because of its hydrophilicity and capacity for nutrient diffusion, the scaffold promoted PDLSC adhesion and proliferation in vitro, allowing cells to migrate into its three-dimensional structure. In comparison to cell-free scaffolds and the untreated group, significant new bone formation (4.43 ± 0.22 mm height, 7.76 ± 0.24 mm^2^ area) and periodontal ligament regeneration (4.25 ± 0.33 mm) were seen in vivo upon implantation into canine mandibular molar defects. These results are associated with the scaffold’s capacity to maintain space for tissue regeneration, combined with the osteoconductivity of HA/β-TCP and the function of β-GP in the development of mineralized nodules [105]. In addition, compared to the other groups, cell-seeded scaffolds increased growth factors (IGF-1, TGF-β, VEGF) and decreased inflammatory cytokines (IL-2, IL-6, TNF-α, TNF-β, INF-γ) due to immunomodulatory factors secreted by PDLSCs that inhibited inflammation and stimulated angiogenesis [106]. Histological analysis demonstrated the scaffold’s bioactivity and degradability by confirming increased osteoblast activity and scaffold integration in cell-seeded scaffolds, with new bone gradually replacing HA/β-TCP remnants. Additionally, the scaffold’s function as a physical barrier that stabilizes the gingival flap and permits true periodontal regeneration was linked to its ability to prevent junctional epithelium invasion (1.05 ± 0.22 mm vs. 2.42 ± 0.14 mm in controls) [69]. These results highlight the scaffold’s dual role as a bioactive platform and structural support, which is consistent with periodontal tissue engineering’s objective of reestablishing 3D architecture and function.

In another study, autologous PDLSC sheets with RGD-modified chitosan scaffolds were transplanted to monkey periodontal defects [74]. It was shown that chitosan scaffolds could provide essential support for cell sheets in periodontal regeneration in the critical size defect. Additionally, RGD peptide, as an important binding motif for a particular transmembrane protein that helps cells adhere to the different ECM proteins, was incorporated into the chitosan scaffold to enhance cellular adhesion, infiltration and proliferation [108]. Superior results were obtained by the PDLSC sheet + RGD-modified chitosan group: the highest alveolar bone density (gray scale value: 21.98 ± 7.85 vs. 7.31 ± 10.27 in chitosan, 16.70 ± 13.17 in RGD-chitosan and 19.34 ± 21.46 in PDL-chitosan), the shortest cement-enamel junction–alveolar bone distance and a 3 mm epithelial attachment gain (compared to 1.75 ± 0.71 mm in chitosan, 2.13 ± 0.83 mm in RGD-chitosan and 2.25 ± 0.71 mm in PDL-chitosan). Furthermore, this group exhibited regenerated periodontal tissues characterized by young osteocytes and enhanced bone formation, as validated by histological analysis and micro-CT imaging [74].

3D scaffolds not only aim to provide biological cues and structural stability for better tissue repair, but also their structural features, like biodegradation and bioabsorption, should match the cells’ regenerative potential to maximize the regeneration process. Collagen is a natural extracellular matrix protein and is widely used in tissue engineering as a biocompatible and biodegradable biopolymer to mimic native tissue structure. Collagen can promote cell adhesion, proliferation and tissue regeneration, making it ideal for applications such as bone, cartilage and skin repair [109]. A study by Matsumura et al. investigated collagen sponge scaffolds seeded with bone marrow mononuclear cells (BM-MNCs) or cortical bone-derived mesenchymal stromal cell (CB-MSC) spheroids to promote bone regeneration around transplanted teeth, simulating alveolar bone expansion in severe combined immunodeficiency (SCID) mice [70]. All groups showed new bone formation in the root furcation area according to micro-computed tomography (μCT) analysis; however, CB-MSC spheroids were the only ones that produced robust ectopic bone formation outside the roots. This result was aligned with the localization of Sp7/osterix-positive osteoprogenitor cells around newly formed bone and prior in vitro findings demonstrating increased expression of osteogenic markers (e.g., ALP, BSP, DMP1) in CB-MSC spheroids compared to BM-MSCs grown in monolayers [110]. It was also noted that relatively rapid degradation of collagen scaffolds (~4 weeks) required highly osteogenic cells, and BM-MNCs, comprising a low volume of stem cells, failed to compensate for it. It was finally demonstrated that collagen fibers were forming interconnections to the new bone and root surface, indicating structural mimicry of periodontal ligament integrity [70].

Recent advancements in clinical studies of cell-seeded scaffolds for periodontal regeneration highlight the powerful synergy between bioactive scaffolds and living cells. In complex defects where natural regeneration is inadequate, researchers hope to achieve more robust healing outcomes by combining these components, where scaffolds offer structural guidance and cells drive tissue repair. To treat human intrabony periodontal defects, autologous alveolar bone marrow MSCs (a-BMMSCs) were seeded into collagen scaffolds along with autologous fibrin/platelet lysate (aFPL) in a clinical study by Apatzidou et al. [71]. Three groups were compared: Group A (a-BMMSCs plus collagen/aFPL), Group B (collagen/aFPL devoid of cells) and Group C (minimal access flap surgery only). There were no intergroup differences in the clinical attachment level (CAL) gain, recession or reduction in probing pocket depth (PPD), and all groups demonstrated significant clinical improvements. However, in contrast to Groups A and C, radiographic bone fill was less noticeable in Group B. At 12 months, Group A defects had a ≥3 mm CAL gain with a PPD of ≤4 mm in 55.6% of cases, compared to 50% in Groups B and C. Additionally, histological analysis verified that Group A had new bone regeneration, whereas 20% of Group B cases showed incomplete flap closure [71]. In another clinical trial, Sanchez et al. seeded autologous periodontal ligament-derived mesenchymal stem cells (PDL-MSCs) in porcine collagen sponges as a xenogeneic bone substitute (XBS) for periodontal regeneration in one- to two-wall intra-bony defects, in comparison to XBS alone [72]. This quasi-randomized controlled pilot phase II clinical trial assigned 20 patients (human in vivo model) necessitating tooth extraction and exhibiting corresponding defects to either the experimental group (XBS + autologous PDL-MSCs derived from extracted teeth) or the control group (XBS alone). Clinical outcomes were assessed over a period of 12 months. The results indicated that the experimental group exhibited a superior mean CAL gain (1.44 mm compared to 0.88 mm) and PPD reduction (2.33 mm versus 2.10 mm); however, the differences were statistically insignificant owing to low power (12.2%) resulting from a small sample size. Safety was validated with no significant adverse events reported. The lack of a significant difference was attributed to limited cell availability in periodontitis patients, the complicated nature of the defects (which had one- to two-wall shapes) and other factors like the depth of the bone and the angle of the defect that could influence the results. The underlying mechanisms may include MSC paracrine effects; however, direct regenerative superiority was not established, requiring larger trials for confirmation [72].

One of the challenges to achieving predictable regeneration of periodontal defects is the junctional epithelium’s apical migration along the exposed root surface, which interferes with and competes with the repopulation of periodontal tissues like cementum, bone and ligament. This rapid epithelial downgrowth often leads to the slower regeneration of supportive structures, thereby hindering natural functional restoration of the periodontium. Thus, evidence indicates that the most prevalent cell type in gingival tissue, gingival fibroblast (GF), migrates to the area of the defect. On the other hand, GF has been shown to be able to form a mineralized matrix, express certain bone-associated proteins and form hard tissue when given the right stimulating conditions [73,111]. Using 3D-engineered scaffolds enables researchers to guide the regenerative potential of cells toward desirable function in complex structures, such as the periodontium. In a clinical study, researchers loaded GF cells and their associated mesenchymal stem cells (GMSC), along with β-tricalcium phosphate (β-TCP) and collagen membrane, into the intrabony periodontal defects. Twenty intrabony defect patients were split into two groups. Group I received β-TCP covered by a non-perforated collagen membrane, while Group II received β-TCP loaded with autologous cultured GF and their associated GMSC, followed by membrane coverage. At six months, Group II showed considerably higher radiographic bone gain (3.14 ± 1.33 mm vs. 1.91 ± 0.16 mm) and a more substantial decrease in CAL gain (6.30 ± 2.06 mm to 2.30 ± 1.16 mm vs. 5.30 ± 0.95 mm to 4.20 ± 1 mm) and vertical pocket depth (VPD: 7.5 ± 2.42 mm to 3.10 ± 0.88 mm vs. 6.50 ± 0.53 mm to 5.20 ± 0.8 mm in Group I). Additionally, on days 1, 3 and 7 following surgery, Group II’s gingival crevicular fluid (GCF) had greater levels of PDGF-BB. Gingival fibroblasts and their associated mesenchymal stem cells’ osteogenic differentiation potential when exposed to periodontal extracellular matrix mediators, as well as their cooperative function as feeder cells boosting MSC viability through growth factor secretion, were responsible for the better clinical and radiographic results in Group II (β-TCP + GF/GMSC) [73,111]. β-TCP’s osteoconductive qualities promoted progenitor cell recruitment and osteogenesis by facilitating calcium ion release and graft remodeling [112]. While decreased BMP-2 levels in Group II at day 14 indicated immunomodulatory suppression of inflammatory bone resorption by GMSCs, elevated PDGF-BB levels in early healing phases indicated enhanced cellular recruitment and angiogenesis [73].

### 3.2. Hydrogels

Hydrogels are polymeric structures with a high amount of water molecules between the polymeric chains. These 3D structures are highly appealing for biomedical applications, particularly tissue engineering, as they replicate the physicochemical architecture of the native extracellular matrix. Hydrogels provide distinct advantages over alternative scaffold types, including tissue mimicry and prolonged drug delivery [104]. Furthermore, for periodontal regeneration, hydrogels can sustain a moist environment similar to that of the oral cavity [113]. Injectable cell-laden hydrogels facilitate minimally invasive, targeted delivery of therapeutic cells to periodontal defects, improving cell viability and integration within complex tissue architectures. Local administration of injectable fibroin/chitosan oligosaccharide lactate hydrogel (F/COS) containing GMSC in rat periodontitis models was studied by Balaban et al. [76]. In comparison to the untreated and hydrogel-only (F/COS) groups, the F/COS + GMSC group showed noticeably more alveolar bone regeneration; micro-CT revealed increased bone volume, and histology revealed well-organized periodontal ligaments. At 8 weeks, the effects of F/COS alone were statistically insignificant, despite the fact that it decreased inflammation and bone loss when compared to the untreated group. Through secreted factors and ECM interactions, GMSCs in the hydrogel enhanced tissue repair by promoting osteogenic differentiation and immunomodulation. Synergistically, the F/COS hydrogel enhanced GMSC viability and offered structural support, promoting regeneration [76].

It is advantageous to load hydrogels with various bioactive agents for proper and improved regeneration. These bioactive agents could be a wide range of materials, such as drugs, growth factors and nanoparticles [104]. Li et al. designed a multifunctional hydrogel with temperature-triggered in situ gelation capacity as a niche for ectomesenchymal stem cells (EMSCs) for periodontitis treatment (Figure 3C) [107]. The material used to form the hydrogel was poloxamer 407 (PX), a triblock copolymer of poly (ethylene glycol)-block-poly (propylene glycol)-block-poly (ethylene glycol). An antioxidative and anti-inflammatory nanoparticle (defined as TPCD) was synthesized from β-cyclodextrin (β-CD), a cyclic oligosaccharide that has good in vivo safety and was added to the hydrogel matrix. Additionally, PX hydrogel was functionalized with an adhesive peptide PPLFMLLKGSTR, which is derived from the globular domain 3 of human laminin-5 α3, to enhance EMSC cell binding, adhesion and survival [107]. The in vivo findings showed that the multifunctional hydrogel (PXN) successfully reduced oxidative stress markers (H_2_O_2_) and pro-inflammatory mediators (TNF-α, IL-1β) in periodontal tissues, thereby normalizing the pathological microenvironment and reducing periodontitis in rats. As demonstrated by the decreased distance of the cementoenamel junction to the alveolar bone crest (CEJ-ABC distance) and improved trabecular bone parameters, including bone volume per tissue volume (BV/TV), trabecular thickness (Tb.Th) and trabecular number (Tb.N), the addition of EMSCs to PXN (PXNE) synergistically improved alveolar bone regeneration. This effect was ascribed to increased EMSC survival and osteogenic differentiation. Superior bone microarchitecture resulted from further functionalization with a laminin-derived peptide (PPNE), which increased EMSC adhesion and viability. By inhibiting the GDF15/Atf3/c-Fos axis of the MAPK pathway, TPCD nanoparticles in the hydrogel reduced inflammation and oxidative stress while fostering osteogenesis [107].

In a study by Zong et al., an injectable collagen hydrogel embedded with hPDLSCs pretreated with gold nanocomplexes (AuNCs) was designed to prevent the resorption of alveolar bone [77]. Exhibiting low cytotoxicity, AuNCs were easily absorbed by primary hPDLSCs and successfully stimulated hPDLSC osteogenic differentiation in vitro. In rat models of orthodontic tooth movement (OTM)-induced alveolar bone resorption, five groups were evaluated: Group 1 (no OTM), Group 2 (OTM only), Group 3 (OTM + sham scaffold), Group 4 (OTM + untreated hPDLSCs) and Group 5 (OTM + AuNCs-treated hPDLSCs). Groups 2–4 showed significant alveolar bone loss, as shown by increased trabecular separation (Tb.Sp) and decreased bone mineral density (BMD, g/cm^3^), BV/TV and Tb.N, according to micro-CT analysis. In contrast, Group 5 showed Tb.Sp. and BMD that were preserved, similar to Group 1. These results may be explained by the transplantation of AuNC-treated hPDLSCs (Group 5), which immunohistochemically revealed decreased osteoclast activity (↓RANKL/osteoprotegerin) and increased osteoid formation, which were associated with upregulated autophagy (↑LC3, Beclin1; ↓P62) [77,114]. AuNCs activated autophagy-mediated osteogenesis, while the type-I collagen scaffold supported cell viability, preventing alveolar bone resorption without affecting OTM [77].

In another study, Chang et al. developed a collagen hydrogel laden with human periodontal ligament fibroblasts (HPLFs), containing riboflavin, with a safe photo-cross-linking process using a dental light-emitting diode (LED) for periodontal regeneration [78]. After transplantation to the rat model with artificial periodontal defects, the COL_HPLF_LED group showed significantly reduced relative epithelial downgrowth and residual bone defects after 6 weeks compared to the Blank, COL_LED and COL_HPLF groups. These results may be the consequence of both the presence of HPLF cells as providers of regenerative and signaling cues and a stiffer matrix due to crosslinking, which resulted in the function of structure as a barrier for clinically guided tissue regeneration, allowing for the growth of bone and inhibiting epithelial downgrowth. It was shown that collagen scaffold stiffness was increased by LED photo-cross-linking, providing structural support during biting and surgery. In vitro, HPLFs developed into myofibroblasts and pre-osteoblasts, and in vivo, they secreted regulatory factors that facilitated osteoblast recruitment and guided the regeneration of the PDL. Functional periodontal regeneration was demonstrated by the combined mechanical stability (through riboflavin-LED cross-linking and HPLF-mediated signaling), which reduced epithelial migration and improved alveolar bone repair [78].

Gelatin methacrylate (GelMA) is one of the most promising hydrogels for biomedical applications and tissue regeneration. GelMA is a gelatin derivative that is highly desirable to researchers because of its strong affinity for native mammalian extracellular matrix and its ease of crosslinking. GelMA comprises a significant amount of methacrylamide groups and a lower proportion of methacrylate groups, which can undergo crosslinking via radical polymerization in the presence of a photo-initiator upon ultraviolet exposure [53]. In a study by An et al., GelMA hydrogels containing hPDLSC sheets and two different types of graphene oxide quantum dots (GOQDs) were used to treat a periodontal rat defect model (Figure 3B) [75]. The results of the study showed that hydrogels embedded with GOQDs with more oxygen-containing groups (Y-GOQD) showed enhanced osteogenic differentiation of hPDLSCs compared to the other group (containing B-GOQD). Furthermore, in comparison to B-GOQDs, hydrogels containing Y-GOQDs induced strong extracellular matrix formation by hPDLSC sheets and significantly restored rat mandibular bone defects, with higher bone volume (BV/TV) and mature bone regeneration [75]. These results are associated with the zero-dimensionality of GOQDs that enables them to easily pass through cell membranes without causing any damage to the lipid membrane structure [115]. Y-GOQDs’ oxygen-rich functional groups (hydroxyl and epoxy bonds) improve the adsorption of osteogenic inducers (like dexamethasone and ascorbic acid) to boost signaling pathways (like Wnt/β-catenin and Col-1 synthesis) and control mitochondrial dynamics by promoting fusion (↑MFN2, OPA1) and preventing fission (↓DRP1, MFF, FIS1), which causes cellular metabolism to shift toward oxidative phosphorylation to meet osteogenic energy demands [75].

More intricate fabrication techniques can be employed to improve the structural features of the construct toward optimal synergistic regenerative capacity. Wang et al. used the freeze-casting technique to design an aligned porous cell containing a hydrogel scaffold based on chitosan (CS) and oxidized chondroitin sulfate (OCS) to induce the arrangement of periodontal tissue regeneration (Figure 3D) [79]. Aligned porous hydrogel (A-HY) treatment groups with periodontal ligament stem cells (PDLSC/A-HY) or gingival mesenchymal stem cells (GMSC/A-HY) showed an improved bone regeneration (micro-CT: BV/TV ~75.39% and 73.63%, respectively) in a rat periodontal defect model compared to disordered pore hydrogel (PDLSC/D-HY: 62.53%) and A-HY alone (55.33%). According to histological analysis, the scaffold’s aligned pore structure (90.13 ± 24.52 μm) improved cell alignment and nutrient exchange, resulting in organized periodontal ligament formation in aligned hydrogel groups [79]. These results are consistent with earlier research showing that scaffolds with a large number of uniformly sized pores facilitate faster cell colonization [116]. Additionally, disordered hydrogels are thought to have impacted the stem cells’ ability to regenerate by decreasing the diffusion of nutrients, oxygen and cellular waste. Thus, regulating the hydrogel’s pore size and direction are important for the regeneration and repair of periodontal tissue defects, particularly those involving the periodontal ligament [79].

The composition of bioengineered structures can modulate cellular behavior. PDLCs can demonstrate two principal functions: alkaline phosphatase (ALP) activity and matrix mineralization, both of which can be modulated to enhance periodontal regeneration. In a study, Fraser et al. investigated the regenerative potential of dual peptide-functionalized poly (ethylene glycol) (PEG) hydrogels, incorporating RGD (arginine-glycine-aspartic acid) and GFOGER (glycine-phenylalanine-hydroxyproline-glycine-glutamate-arginine) to control the behavior of PDLCs and promote the healing of periodontal tissue [80]. Given the complexity of periodontal interfaces, the study showed that scaffold design can independently adjust these processes to regenerate mineralized tissues (cementum/bone) or fibrous PDL-like tissues (high ALP) [80,117]. ALP-optimized hydrogels (high RGD/low GFOGER) mimicked the non-mineralized PDL by increasing alkaline phosphatase (ALP) activity while preventing mineralization through sustained pyrophosphate (PPi) levels [117]. In contrast, hydrogels optimized for mineralization (moderate RGD/high GFOGER) increased osteogenic genes (SP7, IBSP) to promote mineralization, decreased pyrophosphate (PPi) inhibition and activated integrin α2β1-FAK signaling [80]. While ALP-optimized hydrogels promoted fibrous PDL-like repair and cementum formation (45–75% coverage), mineralization-optimized hydrogels significantly increased mineral density and bone regeneration (56% defect fill vs. untreated) in vivo. Despite their persistence close to regenerated tissues, transplanted PDLCs indirectly facilitated repair, most likely by controlling host cells paracrinely [80]. These results demonstrate how dual-peptide hydrogels use integrin-specific extracellular matrix cues to spatially control the fate of PDLC, providing a tunable approach to multiphasic periodontal tissue regeneration [80].

Adhesion to the defect site is a critical concern that bioengineered tissues are expected to resolve to enhance the regenerative function of transplanted cells. Hasani-Sadrabadi et al. addressed the drawbacks of poor adhesion and inadequate osteoconductivity in craniofacial bone regeneration by creating a bioinspired, photocrosslinkable adhesive hydrogel from methacrylated alginate functionalized with dopamine [81]. The hydrogel, which contained hydroxyapatite microparticles and GMSC aggregates, showed good adherence to oral tissues in moist conditions because of dopamine-mediated hydrogen bonding and mussel-inspired interfacial interactions. It was shown that GMSCs exhibited enhanced osteogenic gene expression (RUNX2, OCN) and mineralization, attributed to HAp’s osteoinductive properties, in vitro [118]. In a rat peri-implantitis model, due to improved cell–cell signaling in aggregates, the adhesive hydrogel loaded with GMSC aggregates and HAp surpassed cell-only formulations in achieving full bone regeneration around infected implants [119]. Moreover, the visible-light crosslinking of the hydrogel allowed for accurate defect filling, and medium-degree dopamine conjugation maximized adhesion strength (beyond fibrin glue) without affecting mechanical stability [81,120].

Hydrogels, a versatile periodontal regeneration platform, are biocompatible, extracellular matrix-mimicking and adjustable for drug delivery and cell encapsulation. Their injectability, photo-crosslinkability and ability to retain bioactive agents (e.g., stem cells, nanoparticles, peptides) enhance spatial control over tissue repair, as demonstrated by enhanced osteogenesis and immunomodulation in preclinical models. However, there remain challenges, such as lack of mechanical strength to support loads, dependency on complex functionalization to optimize bioactivity and possible inconsistencies in crosslinking efficiency. A potential strategy to resolve this issue involves simplifying the fabrication process by integrating versatile bioactive agents with multifunctional properties (e.g., adhesive, mechanical strength, anti-inflammatory and angiogenic effects), such as nanoparticles, rather than employing multiple bioactive agents simultaneously. Furthermore, hydrogels in periodontal regeneration face the dual challenge of spatially controlling tissue formation, promoting osteogenesis and cementogenesis at one interface while simultaneously preventing epithelial downgrowth and facilitating connective tissue repair at the other. This “double-faced” functionality requires precise architectural and biochemical cues to balance heterogeneous regeneration (bone, cementum, oriented PDL fibers) against soft tissue competition. Furthermore, hydrogels must conform to the unique geometries of patient-specific defects. Translating small-animal successes to human-scale defects demands scalable designs, dynamic degradation and dual-sided bioactive delivery to synchronize multi-tissue healing in complex periodontal microenvironments. More advanced spatio-biochemical designs require more sophisticated and high-tech methods, such as 3D bioprinting, which allow for enhanced control over the structural characteristics of hydrogels to tackle the complexities of the periodontium.

### 3.3. Three-Dimensional Bioprinting

Three-dimensional printing is a computer-assisted technique in regenerative medicine that has garnered significant attention over the past decade for various tissue engineering applications due to its capacity to accurately design intricate geometries [121]. Three dominant 3D printing technologies include inkjet-based, extrusion-based and light-based methods of fabricating biomedical structures. Inkjet printing utilizes piezoelectric or thermal actuation to deposit biomaterial droplets in a layer-by-layer fashion, providing rapidity and accuracy. Extrusion-based printing employs mechanical and pneumatic forces to extrude bioinks, valued for their compatibility with various materials and gentle processing conditions. Light-based methods utilize geometric patterns (through UV, laser or visible light) to photopolymerize hydrogel precursors, facilitating complex 3D structures [104,121]. These techniques evolve into bioprinting platforms when combined with cell-laden bioinks, facilitating the integration of growth factors and co-culture systems for tissue-specific regeneration [121].

Compared to conventional bioengineering methods that are unable to accurately replicate the complexity of the structure, 3D bioprinting might be a beneficial way to address the intricate and irreversible structural and functional breakdown of the periodontium [82]. In a study by Ma et al., biomimetic peridontium patches (BPPs) containing dental follicle cells (DFCs) were fabricated via microscale continuous digital light projection (mCDLP) bioprinting (Figure 3E) [82]. BPPs aimed to recapitulate the functional “sandwich structure” of the periodontium: alveolar bone, cementum and oriented PDL fibers. BPPs with GelMA-based bioinks were created with columnar hydrogel pillar structures (resembling PDL fiber orientation) and grid supports for stability. The precise spatial guidance for DFCs was made possible by mCDLP bioprinting, and the porosity and low viscosity of GelMA guaranteed cell viability and nutrient diffusion, which promoted collagen maturation and ECM deposition. Four groups were tested in periodontal defect models in rats and beagle dogs: blank (defect without treatment), fiber-oriented (BPP-implanted), random (disordered hydrogel blocks) and native (no defect). The findings demonstrated that in fiber-oriented groups, thanks to the biomimetic microarchitecture guiding DFC alignment and differentiation into ligament-forming cells, BPPs completely restored the alveolar bone-PDL-cementum complex; PDL fibers displayed region-specific orientations (oblique, horizontal and alveolar crest groups) [82,122]. In contrast, in random and blank groups, they displayed disordered fibers or incomplete healing. Additionally, BPPs stimulated DFC differentiation and collagen maturation, upregulated osteoprotegerin and inhibited RANKL-mediated osteoclast activation to improve bone regeneration [82].

Using an extrusion-based bioprinter, Miao et al. developed a versatile 3D-bioprinted scaffold using a hybrid hydrogel of GelMA, sodium alginate and bioactive glass microspheres (BGM) to regenerate both periodontal soft (gingiva) and hard (alveolar bone) tissues [83]. The findings demonstrated that BGM improved osteogenic differentiation of bone marrow mesenchymal stem cells (upregulated ALP, OPN and Runx2), mechanical stability and apatite formation (through Si/Ca/P ion release). Cell viability, proliferation and fibronectin expression were all enhanced by the dual-functional scaffolds (BMP2/PDGF) for soft tissue repair. Within eight weeks, these scaffolds restored gingival tissue (↓probing depth) and alveolar bone (↑BV/TV, trabecular number) in Beagle dog defects. The hydrogel’s ECM-like structure, growth factor synergy and BGM-driven mineralization were important mechanisms that supported tissue integration and cell survival [83].

Different bioprinters may exhibit distinct characteristics, and their combination could facilitate the precise engineering of structures for complex sites like the periodontium. To overcome restrictions in bone-ligament interface fusion and fiber orientation, Yang et al. fabricated 3D bioprinted composites with two different bioprinting techniques for periodontal regeneration. Bioinks were made of GelMA with dental follicle-derived decellularized ECM containing human DFCs [84]. Digital light projection (DLP) for the PDL module and direct ink writing for the alveolar bone module were the two bioprinting methods used in the study, which were specifically designed to meet the unique requirements of periodontal regeneration. With its high resolution (~150 μm pillars), DLP was able to guide dental follicle cells into oriented arrangements that are necessary for ligament regeneration by creating precise, lattice-like structures that mimic natural fiber alignment. On the other hand, direct ink writing extrusion-based method produced a strong, porous grid (410 μm strands) for the bone module, supporting osteogenesis by striking a balance between nutrient diffusion and mechanical stability. This smart combination met the two anatomical needs of the periodontal complex by utilizing direct ink writing structural adaptability for hard-tissue regeneration and DLP’s accuracy for soft-tissue guidance [84]. In vivo, the bioprinted structure promoted aligned PDL fibers, highly mineralized alveolar bone and functional bone–ligament interfaces in beagle defects, alongside reduced inflammation via decellularized ECM-mediated suppression of M1 macrophage activation. These results are correlated with the bioprinted architecture providing topographical guidance for fiber alignment and the retention of tissue-specific biochemical cues (sGAG, collagen) by decellularized ECM, which promoted cell behavior and differentiation. The immunomodulatory activity of decellularized ECM, attributed to retained ECM components, mitigated proinflammatory responses, enabling integrated tissue regeneration without immune rejection [84,123,124].

The application of bioprinters should match the target tissue and represent an optimal selection for bioink composition. Dubey et al. used microvalve-based bioprinting (in the inkjet category) for its precise spatial control over bioink deposition and minimal shear stress while enabling the fabrication of complex 3D constructs with uniform amorphous magnesium phosphate (AMP) particle distribution [86]. Authors hypothesized that the integration of amorphous magnesium phosphate (AMP) into an ECM-based hydrogel bioink would improve the osteogenic differentiation of hDPSCs and promote bone regeneration independently of exogenous growth factors, thereby overcoming the drawbacks associated with slow-resorbing calcium phosphate bioceramics and reliance on growth factors [86]. To evaluate this, they formulated a bioink that integrates a synthetic ECM hydrogel (2% octapeptide FEEFEKFK, 98% water) with AMP particles (0.5% or 1.0% *w*/*w*) to leverage AMP’s superior solubility, Mg^2+^ release and ECM-mimetic architecture. The results indicated a high viability of hDPSCs (~90%) and an elongated morphology in AMP-modified bioinks, which is associated with the surface topography of AMP-facilitated cell attachment. Furthermore, AMP induced osteogenic differentiation through the release of Mg^2+^, as demonstrated by increased alkaline phosphatase activity, mineralization and upregulation of RUNX2, COL1A1 and OPN gene expression in the absence of osteogenic media [125]. In vivo, ECM/AMP demonstrated 1.7- and 1.4-fold improvement in bone volume/total volume (BV/TV) at 4 and 8 weeks, respectively, relative to AMP-free ECM, with higher bone density associated with Mg^2+^-mediated osteoblast activation and mineral mobilization. Accelerated AMP resorption enhanced cellular infiltration and mineralization, while the ECM hydrogel offered structural support similar to native tissue, synergistically advancing bone maturation and defect bridging [86].

Hybrid frameworks can also be utilized via 3D printing to attain improved structural support alongside the osteointegration characteristics of bioprinted scaffolds. Lee et al. hypothesized that bioprinting hPDLSCs onto 3D-printed titanium scaffolds could facilitate the regeneration of PDL tissue, thereby mitigating infection susceptibility and mechanical instability surrounding dental implants resulting from PDL deficiency [85]. They developed a hybrid artificial organ by integrating electron beam melting-fabricated porous titanium scaffolds with bioprinted human PDLSCs encapsulated in collagen-based bioink ± FGF-2. Then, the bioprinting group was then compared with cell seeding groups for periodontal regeneration. The cell seeding groups (G1, G2) printed acellular collagen or collagen/FGF-2 inks onto titanium scaffolds, then manually seeded hPDLSCs after gelation. On the other hand, the bioprinting groups (G3, G4) printed bioink that had been mixed with hPDLSCs (±FGF-2) directly onto scaffolds. In athymic rat mandibular defect models, G3 and G4 demonstrated organized periodontal ligament-like connective tissue between the scaffold and bone, expressing periostin, HLA, vWF and CEMP1, whereas G1 and G2 exhibited osseointegration without periodontal ligament formation. The outcomes were ascribed to accurate cell alignment, elevated CEMP1 expression (a cementogenic marker) and uniform cell distribution in bioprinted groups, facilitated by regulated spatial arrangement of hPDLSCs, fostering fibrous tissue regeneration that emulates native PDL functionality [85].

3D bioprinting presents significant potential for periodontal regeneration by facilitating the accurate creation of biomimetic, multi-tissue structures that accommodate the structural intricacies of the periodontium. Methods such as extrusion-based, light-based and hybrid bioprinting enable spatial regulation of cell-laden bioinks (e.g., GelMA, decellularized ECM) and bioactive signals (Mg^2+^, FGF-2), promoting region-specific differentiation into alveolar bone, cementum and oriented periodontal ligament fibers. Preclinical studies indicate efficacy in restoring functional interfaces and suppressing osteoclast activity via architectural guidance (e.g., pillar arrays, lattice grids) and immunomodulatory bioinks. Nonetheless, obstacles remain in addressing human-sized defects, guaranteeing mechanical durability under masticatory forces and attaining prolonged integration with host tissues. Future developments in hybrid bioinks, dynamic degradation profiles, utilizing in situ bioprinting, and clinical validation will be essential for realizing the complete potential of 3D bioprinting in personalized, functional periodontal reconstruction.

### 3.4. Decellularized Extracellular Matrix

Decellularized ECM have arisen as advantageous scaffold materials in periodontal tissue engineering owing to their intrinsic biocompatibility, structural integrity and capacity to replicate native tissue microenvironments [126]. The decellularized human amniotic membrane (DAM), sourced from the innermost layer of the placenta, presents distinct benefits, such as immune privilege, a collagen-rich structure and osteoconductive characteristics. DAM acts as a bioactive scaffold that facilitates cell adhesion, proliferation and differentiation while promoting vascularization and integration with host tissues [91]. Dziedzic et al. investigated the application of a DAM in conjunction with adipose-derived stromal cells (ADSCs) and a mineralized ECM produced in vitro by ADSCs for periodontal regeneration [91]. The decellularized DAM, treated with sodium dodecyl sulfate, maintained its collagen structure and facilitated the adhesion, proliferation and osteogenic differentiation of ADSCs, resulting in a mineralized ECM. Four groups of treatments were tested on rat models with periodontal furcation defects: DAM alone (T1), DAM with ADSCs (T2), DAM with ECM (T3) and DAM with both ECM and ADSCs (T4), compared to untreated defects (T0). After four weeks, micro-CT and histological analysis demonstrated that T1 and T2 significantly improved bone regeneration at the gingival level due to DAM’s osteoconductive facilitation of endogenous cell migration. T3 and T4 demonstrated limited effectiveness owing to increased scaffold rigidity resulting from ECM mineralization, which hindered surgical site closure. Histologically, all treatments facilitated periodontal ligament regeneration, characterized by perpendicular collagen fibers connecting new cementum-like tissue and bone, whereas untreated defects displayed endogenous healing as well. DAM integrated easily, enhancing vascularization without inducing inflammation; however, its physical constraints in ECM-loaded groups suggested the necessity for scaffold adaptability [91].

### 3.5. Microsphere

Microspheres have arisen as adaptable vehicles for stem cell administration in periodontal regeneration owing to their injectability, adjustable porosity and ability to replicate the extracellular matrix. Among synthetic polymers, poly (lactic-co-glycolic acid) (PLGA) has been extensively investigated, whereas naturally derived materials such as gelatin methacryloyl (GelMA) provide bioactivity and biocompatibility. Recent studies have improved microsphere designs by integrating bioactive coatings, antimicrobial agents or hypoxia-mimicking factors to enhance stem cell efficacy in the inflammatory periodontal microenvironment. Liu et al. created PLGA porous microspheres (PMs) coated with silk fibroin (SF) and hydroxyapatite (HA) to deliver hPDLSCs using a “cell perfusion” method (Figure 3F) [90]. The SF coating enhanced hydrophilicity and cellular adhesion, whereas HA offered osteoinductive signals. In vivo, HA-SF-PLGA microspheres surpassed unmodified PLGA and single-component hybrids in a rat periodontitis model, exhibiting reduced cementoenamel junction-alveolar bone crest (CEJ-ABC) distances and enhanced bone mineral density. This result was ascribed to SF-facilitated cell retention and HA-induced osteogenic differentiation, which collectively improved alveolar bone repair [90].

In another study, Luo et al. investigated inflammation-induced stem cell dysfunction by designing PLGA/polydopamine/CGRP microspheres, in which polydopamine improved cell adhesion and facilitated the sustained release of calcitonin gene-related peptide (CGRP) [88]. CGRP inhibited the activation of the ROS/NLRP3/Caspase-1 pathway in BMSCs, thereby reducing inflammation and apoptosis. In vivo, these microspheres diminished osteoclast activity and facilitated alveolar bone regeneration in mice, resulting in notable enhancements in BV/TV and BMD, highlighting the dual functions of polydopamine in cellular protection and the immunomodulatory effects of CGRP [88]. GelMA microspheres also were recently investigated by Chen et al. in a rat periodontitis model [87]. The study introduced hypoxia-preconditioned GelMA-antimicrobial peptide (AMP) microspheres that encapsulate hypoxia-inducible factor (HIF-1α) and stem cells derived from human exfoliated deciduous teeth. HIF-1α promoted SHED proliferation and angiogenesis, while AMP controlled infections locally. In a rat periodontitis model, HIF-1α@GelMA-AMP@SHED microspheres diminished pro-inflammatory cytokines (e.g., IL-6, TNF-α) and enhanced vascularization, resulting in significant alveolar bone regeneration. This success resulted from AMP-induced bacterial resistance and HIF-1α’s capacity to replicate hypoxic conditions, enhancing SHED survival and paracrine function [87]. These studies collectively emphasize the significance of material modifications (e.g., SF, polydopamine), bioactive loading (e.g., HA, CGRP) and microenvironmental regulation (e.g., hypoxia mimicry, antimicrobial activity) in the development of microsphere systems aimed at mitigating periodontal inflammation, improving stem cell functionality and promoting tissue regeneration.

### 3.6. Gold Nanoparticles in Gene-Modified Periodontal Regeneration

In recent years, certain functional bio-nanomaterials have been employed in bioengineering and tissue engineering. Research on nanoparticles primarily focuses on tissue engineering, drug delivery, gene delivery and cell labeling [127]. Gold nanoparticles (AuNPs) are acknowledged as optimal inorganic nanomaterials for biomedical applications, such as drug delivery, diagnostic imaging and targeted therapy, owing to their superior biocompatibility and adaptability in surface modification [89]. For decades, nanotechnology, including nanofabrication, nanofiltration and synergistic therapy, has demonstrated an inseparable connection with viral biology. Li et al. conducted a study utilizing AuNPs conjugated with adenovirus-mediated human β-defensin 3 for the genetic modification of periodontal PDLCs to improve their osteogenic differentiation for periodontal regeneration [89]. The results indicated that AuNPs synergistically enhanced osteogenic markers (ALP, Runx2, COL1) in hPDLCs. In vivo showed rat PDLCs treated with AuNPs diminished alveolar bone loss in rat periodontal defects, resulting in increased BMD and BV in the Ad-human β-defensin 3 + AuNPs group. AuNPs activated the p38 MAPK pathway, as indicated by increased p-p38 levels, which modulated osteogenesis. The augmented effects were ascribed to AuNPs enhancing gene transfection efficiency and intensifying p38 signaling, thereby facilitating regeneration in inflammatory microenvironments [89].

Biomaterial-assisted cell transplantation has revolutionized periodontal regeneration, with 3D bioprinting emerging as the most promising approach due to its precision in fabricating patient-specific, multi-tissue structures (e.g., aligned bone, cementum and ligament fibers). While freeze-dried scaffolds, hydrogels and decellularized matrices offer simplicity or injectability, 3D bioprinting’s ability to spatially control bioactive cues and cell-laden bioinks enables functional integration of complex periodontal interfaces. Challenges remain in scaling to human defects, ensuring mechanical resilience under masticatory forces and achieving complete host-tissue compatibility. Future developments will depend on hybrid bioinks that combine gene-modified stem cells, dynamic degradation systems and multistructure constructs to improve regeneration in inflammatory environments. Innovations such as machine learning-based design and smart stimuli-responsive biomaterials, along with versatile bioactive agents, could improve the results. For clinical translation, thorough validation of safety, reproducibility and cost-effectiveness, along with standardized metrics in human trials, will connect preclinical success to real-world application. By integrating material innovation with biological insights, 3D bioprinting and related strategies are set to provide reliable, functional periodontal restoration, meeting the complex structural and biological requirements of the periodontium.

## 4. Delivery Methods for Biofabrication Products for Periodontal Regeneration

Biofabrication describes the process of constructing living tissues and organs from cells and biomaterials [128]. The field of biofabrication has matured from early scaffold-based tissue engineering into manufacturing utilizing tissue self-assembly, bioassembly and bioprinting to produce patient-matched constructs [128,129,130]. Conventional approaches seed cells into biodegradable scaffolds or hydrogels and rely on weeks of culture in bioreactors for in vitro maturation, followed by implantation. However, more recent approaches utilize multicellular spheroids or aggregates as modular building blocks that are layered by computer-aided deposition and allowed to fuse and mature following natural histogenesis and organogenesis rules [129,131,132,133,134,135]. These materials and precision fabrication advances have enabled customized constructs tailored to patients’ anatomy and cellular components, aligning with the growing demand for personalized medicine [136,137]. Irrespective of the approach, biofabrication prioritizes reliable, controllable production pipelines and downstream handling strategies that preserve structural integrity, viability and function from benchtop manufacture through clinical delivery [137,138].

In regenerating degraded supporting tissues around the teeth, moving from biofabrication to clinical use requires maximum attention to how biofabricated constructs are preserved, transported, handled and delivered to the patient [128,138,139]. Therefore, delivery methods of biofabrication products must be considered alongside design choices (scaffold vs. scaffold-free), material properties and manufacturing techniques (bioprinting vs. assembly), given that delivery methods influence viability, structural integrity, sterility and implantation [128,129,130]. In this part of the review, delivery methods refer to how the biofabricated products are delivered or applied to the intended environment (patient, lab setup or device). The primary focus is on obtaining the biofabricated products from the manufacturing stage into their functional context without damage or contamination [140]. Whether these constructs are transferred by manual manipulation, semi-automated injectors or robot-assisted placement, they impose specific requirements for packaging, preservation and integration [138,141,142]. The following subsections describe the tools, advantages and disadvantages of the different delivery methods. Finally, a comparison across methods, economic aspects and analysis of suitable methods for specific materials or applications is reviewed.

### 4.1. Manual Delivery Methods for Biofabrication Products

Manual delivery of biofabricated products is a principal lab-scale approach for fabricating periodontal constructs and testing decellularized ECM-based hydrogels in in vitro and small-animal models [143,144]. Simple hand tools, including spatulas for transfer and spreading, pipettes for dome-deposition and spheroid placement and forceps for handling cell sheets and grafts, are used to move and position cell-laden gels, sheets and small constructs [144,145]. Low-tech bench procedures, such as dome-positioning of cell-laden gels, mold-casting, pipetting of organoids or spheroids into wells, hanging-drop formation, hydrogel-filled microwells and cell-sheet transfer, are used to produce aggregates and short-term 3D cultures [135,138,145,146]. These manual workflows are widely applied to periodontal cells and remain central in many labs. For instance, hPDLSCs and other periodontal progenitors are routinely cultured as spheroids (agarose/microwell or hanging-drop methods) and then embedded in collagen or dental/tissue-derived decellularized ECM gels, with multiple studies reporting preserved stemness and enhanced cementogenic differentiation in spheroid formats [147,148,149].

Manual delivery methods in biofabrication have been proven beneficial, with the primary advantages being accessibility, low capital cost and material flexibility [141]. Manual casting and pipetting are preferred for short-term biochemical and functional assays or when using viscous, low-yield-strength gels because they permit materials that may collapse under some automated extrusion processes and enable quick prototyping of small, multiphasic assemblies [145,146]. Similarly, bench setups allow for complex decellularized ECM blends, viscous hydrogels and rapid iteration of layered (gingiva/PDL/bone) prototypes that are difficult to implement initially with some printers [144,145]. These features support precision, permit fine adjustments and positioning that are useful in small-defect animal studies and custom in vitro assays, enabling experiments impractical to robotise at early stages of method development [145,150].

However, manual delivery methods are time-consuming, labor-intensive and operator-dependent. This often results in sample-to-sample variability, inconsistencies between samples and a higher risk of user error or contamination, limiting the replicability of studies and tissue engineering workflows [141,145,146]. In addition, manual methods struggle with precise cell positioning or layer-by-layer registration and scale poorly to the manufacturing volumes required to recreate the multiphasic periodontium [151,152]. The reproducibility and workflow limitations are widely noted in organoid and 3D culture fields and are among the reasons recent periodontal biofabrication works are moving toward semi-automated or fully automated assembly systems [145,151,153].

### 4.2. Semi-Automatic Delivery Methods for Biofabrication Products

Semi-automatic delivery methods of biofabrication use syringe-based extrusion injectors (driven by pneumatic, piston or progressive mechanisms), microwave droplet systems and pick-and-place bioassembly platforms such as the Kenzan microneedle array [152,154] and aspiration-assisted bioprinting [153,155] to enable dose automation and placement while keeping operators in the loop [153,155,156,157,158]. These injectors hold bioinks in closed cartridges and expel them under controlled pressure through a defined nozzle. Studies in periodontal tissue regeneration have reported a wide usage of extrusion bioprinting in fabricating scaffolds and graded constructs for the PDL-alveolar bone-gingiva complex, providing controlled strand spacing, porosity and layer registration that are difficult to achieve with manual pipetting or spatula transfer [158,159,160]. Similarly, semi-automatic pick-and-place delivery methods have enabled scaffold-free assembly from cellular spheroids, providing relevant options for precision in spatial colocalization of periodontal cell types [152,153,154,155,161,162,163]. This trend has been observed in emerging dental models, including 3D-bioprinted periodontal microtissues for PDL-bone interactions and periodontal bioprinting pipelines from ink preparation to deposition [164,165].

Compared to manual methods, semi-automatic injectors have the advantages of increased efficiency, continuously metered strands or droplets and stabilized flow through flow controls [166,167]. Additionally, they reduce build times, provide greater consistency of samples and reduce handling steps, lowering human use errors and contamination risks during assembly [158,166,167]. However, semi-automatic systems face challenges in placement precision at the single aggregate level compared to fully automated workflows [153,154,155,161,162,168]. This is particularly observed when nozzle diameters, hydrogel rheology and shear stress restrict movement across delicate periodontal interfaces [152,153,158]. Finally, the performance of these semi-automatic injectors is often dependent on periodic calibration and maintenance, given that drifts can degrade strand fidelity and pore geometry, which are critical to periodontal construct function [167,169].

### 4.3. Automatic Delivery Methods for Biofabrication Products

Automatic delivery in periodontal biofabrication involves using portable and in situ bioprinters to deposit cell-laden bioinks directly on oral defects [170]. These automatic platforms complement benchtop systems for performing grafts, building on extrusion bioprinting, photopolymerization (digital light processing) and electrospinning techniques to ensure precision, reproducibility and optimal shaping in periodontal architectures [171,172,173,174,175,176]. Extrusion bioprinting fabricates hydrogel constructs from computer-aided design models. It supports high cell densities with >80% viability when shear is controlled, enabling multi-material periodontal constructs and gradients that emulate the PDL–cementum–bone complex [175,176,177,178,179,180]. DLP cures photocrosslinkable decellularized ECM composites layer-by-layer with projected light, offering high speed and controllable mechanics suitable for delicate periodontal lamellae [181]. Similarly, cell electrospinning enables high-throughput fibrous scaffolds with tunable alignment and porosity for guiding tissue regeneration membranes and fibro-ligament interfaces [182]. Thus, in situ and portable printing conforms to complex intraoral geometries without extensive preoperative scanning, improving delivery precision and consistency at the defect sides despite salivary flow and limited access [170]. This review further notes that recent studies have reported bioprinting PDL-bone modules, microfluidic periodontal models and 3D-printed bioactive constructs, increasing the discussion on the architectural control of automatic methods for periodontal management [84,160,165,183].

The advantages of automatic delivery in periodontal regeneration include high precision and complex-geometry capability across intrabony and furcation defects, heterogeneous and multi-cell or material stacking and cell guidance orientation [176,181,182]. In addition, portable and in situ printers increase speed, portability and defect-conforming deposition, reducing handling steps and maintaining spatial fidelity in the confined oral cavity [170]. Despite its numerous advantages, automatic delivery methods of biofabricated products encounter several challenges. First, it requires a high initial capital cost and technical complexity, and specialized operator training and workflow integration in sterile dental settings are needed [176,181]. Second, there is the risk of cytotoxicity from photopolymerizers and a concern for potential UV-induced viability loss in DLP constructs placed near vital periodontal tissues [181]. Furthermore, shear-induced cell damage during extrusion requires careful pressure profiling and rheological tuning, and the scalability of the method often depends on printer setup and multi-cartridge coordination [176,180]. Also, electrospun constructs may require post-fabrication crosslinking to reach periodontal loading demands, and cell density and positioning are harder to control during cell-electrospinning [182,184].

## 5. Discussion

Periodontal regeneration remains an intricate challenge because of the complex anatomical and biological architecture of the periodontium, which includes mineralized (bone, cementum) and soft tissue (gingiva, periodontal ligament) components, each requiring distinct yet synchronized repair. Despite notable progress in cellular and biomaterial-based technologies, no single therapeutic strategy has yet achieved consistent regeneration across all compartments of the periodontium. Future advances will rely on versatile materials, optimized cellular combinations, mechanical conditioning, intelligent data integration and scalable biofabrication platforms that bridge experimental and clinical domains [16,24].

Traditional regenerative systems often combine several materials or agents to provide antibacterial, angiogenic and osteogenic effects simultaneously. However, these combinations increase fabrication complexity, cost and potential for unpredictable interactions between components. A promising trend is the development of versatile biomaterials capable of performing multiple biological functions through intrinsic or engineered properties. Nanoparticles, such as gold, magnesium phosphate or cerium oxide, exemplify this concept: a single material can modulate inflammation, stimulate stem-cell differentiation, promote vascularization and inhibit microbial growth [77,89]. Similarly, hybrid nanocomposites incorporating bioactive ions or responsive moieties can dynamically adapt to the inflammatory and hypoxic conditions typical of periodontal defects [88,185]. The design of multifunctional biomaterials reduces formulation heterogeneity while maintaining targeted activity, leading to simpler, safer and more reproducible constructs. Incorporating these versatile materials into hydrogels, microspheres or 3D-printed matrices could therefore replace multi-agent formulations and create “smart” scaffolds responsive to biochemical cues, mechanical stress and immune signals.

Equally important is the rational selection and combination of cellular sources according to the regenerative objective. Periodontal tissues do not regenerate uniformly—gingival tissue often demonstrates rapid intrinsic repair, whereas alveolar bone and PDL remain the main barriers to complete regeneration. This heterogeneity necessitates designing composite constructs with cell types specialized for each region. For instance, gingival fibroblasts and their associated mesenchymal progenitors can provide early wound closure and immunomodulation, while PDL stem cells and dental follicle cells contribute to oriented fiber and cementum formation [26]. The co-culture of endothelial progenitors or bone marrow-derived stromal cells further enhances angiogenesis and bone integration [41]. Future strategies should therefore focus on controlled co-printing or spatial arrangement of multiple cell types within a single construct, guided by the microarchitectural pattern of the native periodontium [69,82]. Modular assembly of function-specific cellular units may enable the creation of anatomically truthful, load-bearing regenerative grafts [71].

Beyond biochemical and cellular optimization, mechanical stimuli are emerging as decisive regulators of tissue organization and maturation. The periodontium is physiologically subjected to cyclic forces from mastication and occlusion, which influence stem-cell differentiation, extracellular matrix orientation and angiogenesis [34,150]. Integrating mechanical stimulation into regenerative protocols could enhance tissue maturation and ensure mechanical competence of the regenerated periodontium. Dynamic bioreactors and mechanosensitive biomaterials are increasingly used to precondition constructs before implantation, inducing collagen alignment and mineral deposition similar to native tissue. Mechanically active hydrogels and magneto-responsive scaffolds may also allow localized stimulation after implantation [88]. Designing experimental systems that quantify and reproduce physiologic strain patterns, especially in preclinical models, will help establish mechanical benchmarks for future translational studies.

**Figure 4:**
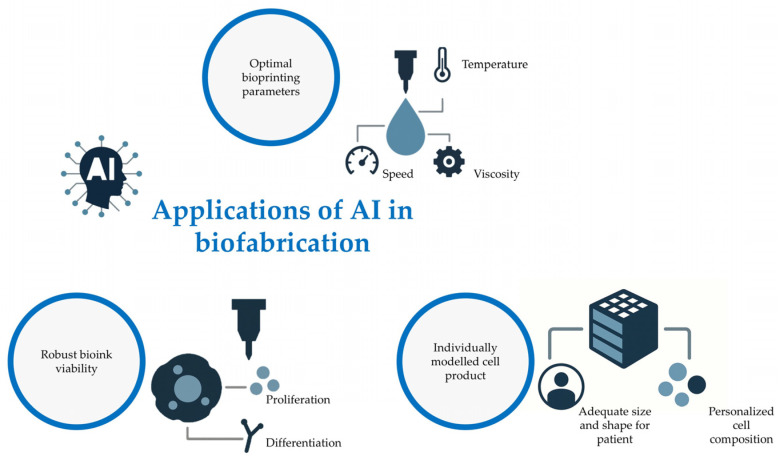
Applications of artificial intelligence (AI) in biofabrication. AI algorithms can optimize bioprinting parameters such as temperature, speed and viscosity; predict bioink viability by modeling cell proliferation and differentiation; and design individualized cell products with personalized size and cellular composition tailored for each patient.

The integration of artificial intelligence (AI) and machine learning into bioengineering research provides a transformative opportunity to analyze complex, multidimensional datasets derived from imaging, molecular assays and biomechanical testing [142]. AI-driven modeling can identify correlations between scaffold composition, cell type and regenerative outcomes, thereby accelerating optimization of formulations and experimental designs (Figure 4). In preclinical and clinical trials, AI-assisted image analysis allows high-throughput quantification of histological and µCT data, minimizing observer bias and improving reproducibility. Predictive algorithms can further support the design of animal studies with optimized sample sizes, minimizing variability and ethical impact. In clinical contexts, machine learning could aid in patient stratification and in monitoring post-implantation remodeling via imaging or biomarker analysis. Establishing standardized digital pipelines linking experimental data with clinical outcomes will be crucial for the evolution of precision regenerative dentistry.

Among all emerging technologies, 3D bioprinting remains the most promising route toward personalized periodontal reconstruction [121,160]. Its ability to spatially position multiple biomaterials and cell types with micrometer precision enables the fabrication of multiphasic, patient-specific constructs that recapitulate the hierarchical structure of bone, PDL and gingiva. However, clinical translation is constrained by challenges in print fidelity, mechanical durability under masticatory forces, sterilization and regulatory standardization [175]. Addressing these challenges will require interdisciplinary innovation, developing bioinks with tunable viscosity and long-term mechanical strength, integrating in situ bioprinting directly within the surgical field and establishing quality control criteria for printed constructs [186]. Hybrid manufacturing that combines conventional scaffolds with localized bioprinting may serve as a bridge toward scalable clinical application [145]. The future of periodontal regeneration will depend on transforming 3D bioprinting from a laboratory prototype to a certified, cost-effective clinical technology.

Although many biofabrication studies emphasize delivery methods (manual, semi-automatic or fully automated) these differences mainly affect workflow scalability rather than the biological efficacy of constructs [171,185]. The overarching goal is not simply to deliver cells or scaffolds efficiently, but to orchestrate the biological, mechanical and digital components into an integrated therapeutic system. The convergence of multifunctional biomaterials, rational cellular design, mechanical conditioning and AI-driven optimization represents a paradigm shift from empirical tissue engineering to predictive and adaptive biofabrication [142].

Despite impressive preclinical progress, the clinical translation of periodontal bioengineering technologies remains limited. Bridging this gap requires standardized protocols for cell sourcing, biomaterial fabrication and delivery methods that comply with good manufacturing practice and medical device regulations. Scalable production platforms, such as automated bioreactors and portable bioprinters, can ensure reproducibility and enable chairside applications. Integration with digital dentistry, including intraoral scanning and computer-aided design of defect-specific constructs, will accelerate individualized therapy. Industrialization efforts must also address cost efficiency, long-term storage and logistics of cell-based materials and regulatory approval pathways for combined advanced therapy medicinal products. Strategic collaboration between academia, industry and clinicians is essential to move from animal validation to human trials, establishing a translational pipeline where biofabricated constructs become accessible and clinically reliable tools for functional periodontal regeneration.

## 6. Conclusions

Periodontal regeneration requires coordinated restoration of bone, ligament and gingiva, which cannot be achieved by conventional methods alone. Recent advances in stem cell therapy, multifunctional biomaterials and biofabrication technologies have brought the field closer to clinically viable, patient-specific solutions. Integrating mechanical conditioning and AI-driven data analysis will further optimize regenerative outcomes and translation. Ultimately, the convergence of smart materials, precise bioprinting and intelligent design frameworks marks the transition of periodontal bioengineering from experimental innovation to practical clinical reality.

## Figures and Tables

**Table 1:** Studies on scaffold-based cell therapy for guided regeneration of periodontal tissues.

Ref	Biomaterials and Additives	Biomaterial Form	Cell Type	In Vivo Model	Main Result
[69]	Chitosan, β-glycerol phosphate, and biphasic calcium phosphate	Freeze-dried scaffold	Canine PDLSCs	Dog periodontal bone defect	Immunomodulatory effects and new periodontal ligament formation in the defect region.
[70]	Collagen	Atelocollagen sponge	BM-MNCs or CB-MSC spheroids	Defect around transplanted tooth in mice	Notable bone formation external to the root was observed in the group of MSCs derived from cortical bone.
[71]	Collagen with aFPL	Porous scaffold	a-BMMSCs	Human intra-bony periodontal defects	The cell-containing scaffolds exhibited a significant reduction in the distance from the cemento-enamel junction to the bottom defect.
[72]	Collagen	Porous scaffold	Autologous PDLMSCs	Human intra-bony periodontal defects	Scaffolds seeded with PDL-MSCs resulted in a marked gain in clinical attachment level and a reduction in probing pocket depth.
[73]	β-TCP and Collagen	β-TCP scaffold covered by a non-perforated collagen membrane	Autogenous GMSCs	Human intra-bony periodontal defects	The cell containing scaffolds demonstrated a decrease in vertical pocket depth and clinical attachment level improvement.
[74]	(RGD)-modified chitosan	Freeze-dried scaffold	PDLs collected from *Macaque* *nemestrina*	*Macaque nemestrina* periodontal defect model	The incorporation of RGD peptide into the chitosan scaffold diminished the distance between the cement-enamel junction and the alveolar bone crest.
[75]	GelMA and GOQDs	Hydrogel	hPDLSCs	Rat periodontitis model	Favorable growth and osteogenic differentiation in vitro, along with enhanced repair of mandibular periodontal bone defects in vivo.
[76]	Fibroin/Chitosan	Hydrogel	Rat GMSCs	Rat periodontitis model	µCT analysis revealed new bone formation in the fibroin/chitosan/GMSC treated group.
[39]	Poloxamer 407 (PX), β-cyclodextrin-based nanoparticle, and PPLFMLLKGSTR adhesive peptide	Hydrogel	EMSCs	Rat periodontitis model	Reduction in cementoenamel junction to the alveolar bone crest distance and improved trabecular bone parameters.
[77]	Collagen and AuNCs for cell pretreatment	Hydrogel	hPDLSCs pretreated with AuNCs	Rat Orthodontic Tooth Movement model	In vitro, AuNCs markedly improved the osteogenic differentiation of hPDLSCs. In vivo, the biomimetic transplantation of AuNCs maintained higher bone mineral density.
[78]	Collagen and Riboflavin	Hydrogel	Human periodontal ligament fibroblasts (HPLFs)	Rat periodontitis model	Significant reduction in epithelial downgrowth, allowing for the growth of bone
[79]	Chitosan and oxidized chondroitin sulfate	Hydrogel	PDLSCs or GMSCs	Rat periodontitis model	The treatment groups of aligned porous hydrogel with PDLSCs or GMSCs demonstrated enhanced bone regeneration.
[80]	PEG functionalized with RGD and GFOGER	Hydrogel	PDLC	Rat periodontal defect	Improved cementum formation in periodontal defects in rats. Hydrogels specifically designed for in vitro mineralization promoted new bone formation.
[81]	Alginate, dopamine, RGD peptide and HAp	Hydrogel	Human GMSCs	Rat Peri-Implantitis Model	Competent adherence to oral tissues in moist conditions because of dopamin-mediated hydrogen bonding and mussel-inspired interfacial interactions. Hydrogels loaded with GMSC aggregates and HAp surpassed cell-only formulations in achieving full bone regeneration around infected implants.
[82]	GelMA	3D Bioprinted structure via DLP bioprinter	DFCs	Rat and beagle dog periodontal defect model	The biomimetic microarchitecture that directed DFC alignment and their development into ligament-forming cells led to a full recovery of the alveolar bone-PDL-cementum complex.
[83]	GelMA, sodium alginate, BGM, BMP2 and PDGF	3D Bioprinted structure via extrusion bioprinting	BMSCs	Beagle dog periodontal defect	BGM improved osteogenic differentiation of bone marrow mesenchymal stem cells and apatite formation.
[84]	GelMA and dental follicle-derived decellularized ECM	3D Bioprinted structure via DLP and direct ink writing bioprinter	Human DFCs	Beagle dog periodontal defect	Promoted aligned PDL fibers in vivo, highly mineralized alveolar bone, and functional bone-ligament interfaces in beagle defects, as well as reduced inflammation.
[85]	Collagen and FGF-2	3D Bioprinted structure via extrusion bioprinting on a titanium 3D printed scaffold	hPDLSCs	Rat mandibular defect model	Organized periodontal ligament-like connective tissue between the scaffold and bone, expressing periostin, HLA, vWF, and CEMP1.
[86]	Commercially available ECM-based hydrogel and magnesium phosphate	3D Bioprinted structure via microvalve-based bioprinting (inkjet-based)	hPDLSCs	Rat calvarial defect model	In vivo, ECM/AMP resulted in higher bone density associated with Mg^2+^-mediated osteoblast activation and mineral mobilization.
[87]	GelMA, HIF-1α, and Antimicrobial peptide	Microspheres	Dental pulp stem cells	Rat periodontitis model	HIF-1α + GelMA + Antimicrobial peptide + Dental pulp stem cells microspheres reduced pro-inflammatory cytokines (e.g., IL-6, TNF-α) and enhanced vascularization, resulting in significant alveolar bone regeneration.
[88]	PLGA, polydopamine and CGRP	Microspheres	BMSCs	Mice periodontitis model	Decreased osteoclast activity and facilitated alveolar bone regeneration, leading to enhancements in bone volume and bone mineralized density
[89]	AuNPs conjugated with adenovirus-mediated human β-defensin 3	Nanoparticles	Human and rat PDLSC	Rat periodontal defect	Diminished alveolar bone loss in rat periodontal defects, resulting in increased bone mineralized density and bone volume.
[90]	PLGA, silk fibroin and hydroxyapatite	Microspheres	hPDLSCs	Rat periodontal defect	HAp-SF-PLGA microspheres surpassed unmodified PLGA exhibiting reduced cementoenamel junction-alveolar bone crest distances and enhanced bone mineral density.
[91]	Human DAM, ASCs and mineralized ECM	Decellularized tissue	ADSCs	Rat periodontal defect	Improved bone regeneration at the gingival level due to and facilitated periodontal ligament regeneration, characterized by perpendicular collagen fibers connecting new cementum-like tissue and bone.

## Data Availability

Not applicable.

## Abbreviations

The following abbreviations are used in this manuscript:

MSCs	mesenchymal stem cells
ASCs	adult stem cells
BMAC	bone marrow aspirate concentrate
SVF	stromal vascular fraction
PDL	periodontal ligament
DPSCs	dental pulp stem cells
PDLSCs	periodontal ligament stem cells
CEMP1	cementum protein-1
CAP	cementum attachment protein
BMSCs	mesenchymal stem cells derived from bone marrow
PDGF	platelet-derived growth factor
FGF	fibroblast growth factor
VEGF	vascular endothelial growth factor
FGF-2	fibroblast growth factor 2
IGF-1	insulin-like growth factor 1
BMSC-sEVs	bone marrow mesenchymal stem cell-derived small extracellular vesicles
TSG101	tumor susceptibility gene 101
hPDLC	human periodontal ligament cells
HSCs	hematopoietic stem cells
SCF	stem cell factor
ECM	extracellular matrix
APC	alveolar periosteal cell
CS	chitosan
β-GP	β-glycerol phosphate
HA/β-TCP	biphasic calcium phosphate
GOQDs	graphene oxide quantum dots
EMSCs	ectomesenchymal stem cells
OCS	oxidized chondroitin sulfate
BPPs	biomimetic periodontium patches
DFCs	dental follicle cells
mCDLP	microscale continuous digital light projection
PMs	porous microspheres
SF	silk fibroin
HA	hydroxyapatite
BM-MNCs	bone marrow mononuclear cells
CB-MSC	cortical bone-derived mesenchymal stromal cell
SCID	severe combined immunodeficiency
μCT	micro-computed tomography
a-BMMSCs	autologous alveolar bone marrow mesenchymal stem cells
aFPL	autologous fibrin/platelet lysate
CAL	clinical attachment level
PPD	probing pocket depth
PDL-MSCs	autologous periodontal ligament-derived mesenchymal stem cells
XBS	xenogeneic bone substitute
GF	gingival fibroblast
GMSC	gingival mesenchymal stem cells
β-TCP	β-tricalcium phosphate
VPD	vertical pocket depth
GCF	gingival crevicular fluid
F/COS	fibroin/chitosan oligosaccharide lactate hydrogel
PX	poloxamer 407
β-CD	β-cyclodextrin
CEJ-ABC distance	distance of the cementoenamel junction to the alveolar bone crest
BV/TV	bone volume per tissue volume
AuNCs	gold nanocomplexes
OTM	orthodontic tooth movement
BMD	bone mineral density
HPLFs	human periodontal ligament fibroblasts
LED	light-emitting diode
GelMA	gelatin methacrylate
PEG	poly(ethylene glycol)
BGM	bioactive glass microspheres
AMP	amorphous magnesium phosphate
DAM	decellularized human amniotic membrane
ADSCs	adipose-derived stromal cells
PLGA	poly(lactic-co-glycolic acid)
CGRP	calcitonin gene-related peptide
HIF-1α	hypoxia-inducible factor 1α
AI	artificial intelligence
